# Robotic excision of complex adrenal mass with retrocaval extension and encasement of renal hilum with renal preservation

**DOI:** 10.1590/S1677-5538.IBJU.2017.0384

**Published:** 2018

**Authors:** Vishnu Raveendran, Ramaprasad Manasseri Koduveli, Kishore Thekke Adiyat

**Affiliations:** 1 Aster Medcity, Kochi, Kerala, India

## Abstract

**Objective:**

The purpose of this video is to present robotic excision of a complex adrenal mass with retrocaval extension and encasement of renal hilum in a 16 year old boy. Biochemical screening was negative for metabolically active component. Computerized tomographic scan with contrast revealed a homogenous mass of approximately 10.8 cm x 6.2 cm x 4.2 cm in the suprarenal area on right side that was extend-ing behind inferior vena cava and encasing renal hilar vessels. Imaging findings were that of a classical ganglioneuroma.

**Material and methods:**

Robot assisted laparoscopic adrenalectomy with sparing of renal hilar vasculature was performed. With patient in lateral position, five ports were used, including one for liver retraction. Da Vinci^®^ system with four arms was docked from over the right shoulder. The displaced renal hilar structures were identified by opening Gerota's fascia. Mass was dissected completely and removed through Pfan-nensteil incision.

**Results:**

Duration of procedure was 345 minutes and console time was 290 minutes. Blood loss was 250 mL. Post-operative renal doppler showed normal blood flow. He was discharged on post-operative day three. Histopathologic examination of specimen revealed ganglioneuroma arising from adrenal gland.

**Conclusion:**

Ganglioneuroma is a rare adrenal tumor with good prognosis on surgical removal. The advent of robotic surgery has made complex surgical procedures involving vital structures like inferior vena cava be performed using minimally invasive techniques without compromising oncologic principles.

## ARTICLE INFO

**Available at: http://www.intbrazjurol.com.br/video-section/20170384_Raveendran_et_al**

**Int Braz J Urol. 2018; 44 (Video #18): 1261-1**

